# Comparison of *Polygonatum sibiricum* Polysaccharides Found in Young and Mature Rhizomes

**DOI:** 10.3390/foods13132010

**Published:** 2024-06-25

**Authors:** Yan Chen, Jing Liu, Yifan Xu, Chaoqun Sun, Wenjie Qu, Hanchen Du, Menglu He, Junsheng Huo, Jing Sun, Jian Huang, Jiyong Yin

**Affiliations:** 1 Key Laboratory of Public Nutrition and Health, National Health Commission of the People’s Republic of China, National Institute for Nutrition and Health, Chinese Center for Disease Control and Prevention, Beijing 100050, China; chenyan7341@163.com (Y.C.); xuyf@ninh.chinacdc.cn (Y.X.); sunchaoqun97@163.com (C.S.); qwj370612@163.com (W.Q.); duhc@ninh.chinacdc.cn (H.D.); heml@ninh.chinacdc.cn (M.H.); huojs@ninh.chinacdc.cn (J.H.); sunjing@ninh.chinacdc.cn (J.S.); huangjian@ninh.chinacdc.cn (J.H.); 2 Aer-Bio Active Health Institute, Beijing 100043, China; liujing@edongfanghong.com

**Keywords:** by-products, extraction rate, structure composition, antioxidant function

## Abstract

The main active component of *Polygonatum sibiricum* (*P. sibiricum*) rhizome is *Polygonatum sibiricum* Polysaccharide (PsP) with antioxidant function. At present, only the mature rhizome of *P. sibiricum* is used to extract PsP, while the young rhizome of by-product is discarded directly as waste, resulting in significant wastage of *P. sibiricum* resources. We used ultrasound-assisted extraction-deep eutectic solvents (UAE-DESs) method to extract PsP of young and mature rhizomes, respectively. The extraction rate, structure composition and antioxidant ability of PsP between young and mature rhizomes were compared, so as to provide references for comprehensive utilization of *P. sibiricum* resources. The PsP extraction rate (33.88 ± 1.95%) of young rhizome was close to that (45.08 ± 1.92%) of mature rhizomes. The main component (PsP-2) of the PsP in young rhizome contained six kinds of monosaccharides, which belonged to acidic polysaccharides. The above characteristics of the PsP of young rhizome were similar to those of mature rhizome. The PsP of young rhizome also exhibited similar biological activity to that of the mature rhizome, which indicated even more advantages in DPPH free radical scavenging ability. The results of this study support the utility of the young rhizome, consequently helping to avoid unnecessary waste and provide reference for comprehensive utilization of *P. sibiricum*.

## 1. Introduction

*Polygonatum sibiricum* (Asparagidae) is one species belonging to the *Polygonatum genus* and a monocotyledonous plant [1], which mainly distributes in temperate regions of the northern hemisphere, such as Japan, Korea, Europe, North America and other countries. It more widely distributes in China, which mainly distributes in forests and bushes in moist and cool environments of the northeast, northwest, north, east, and southwest regions [2]. *P. sibiricum* is a traditional Chinese medicine and food, which has been listed as an edible and medicinal plant in China since 2002 [3]. *P. sibiricum* contains various components, including polysaccharide, steroids, anthraquinone, alkaloids, cardiac glycosides, lignin, vitamins, various acids, and so on [4], and these components can play different functions, respectively [5]. Polygonatum rhizome, as a natural part of medicine and food, has high edible and medicinal value, such as nourishing yin, strengthening spleen, moistening lung and benefiting kidney, and has certain medicinal properties, and few toxic and side effects [6]. It is widely used in the development of clinical and health care products, helping to meet the various health needs of consumers.

Polysaccharides are macromolecular carbohydrates composed of long-chain monosaccharide units connected by glycosidic bonds. Their molecular weights are generally in the tens of thousands or even millions [7,8]. Polysaccharides are an essential component in life activities, and play important roles in cell–cell communication, cell adhesion, and molecular recognition in the immune system [3,9,10]. This has attracted considerable attention from researchers due to their low toxicity and biocompatibility, as well as numerous health benefits [11,12,13].

The rhizome of *Polygonatum* is rich in polysaccharides, which is considered as a new source of nutrients [14]. The rhizomes of *P. sibiricum* together with *P. cyrtonema* and *P. kingianum* are known as “Huangjing” in Chinese medicine. Among them, *P. sibiricum,* which is abundant in the Beijing region, is the main research material in this study. For PsP, the different extraction methods and different types of rhizomes vary in monosaccharide composition, structure, glycosides linkages, functional groups, and molecular weight, which are related to the specific activities [15]. In recent years, the PsP study has been widely concerned. Pharmacological and clinical studies have shown that PsP is mainly composed of galactose (Gal), rhamnose (Rha), and consists of β-2,1- and β-2,6-D-fructosyl bonds, with a molecular weight ranging between 2.2–400 kDa, which belongs to furanose and acidic polysaccharide [2,16,17,18]. In addition, the PsP has many functions, such as immune regulation, anti-tumor activity, anti-inflammatory activity, anti-viral functions, anti-radiation functions, anti-oxidation function, and a hypoglycemic effect [17,19,20].

Different parts of plants have different biological activities and applications due to different structure composition. Previous studies extracted and determined the structure of polysaccharides from different parts of Nelumbo nucifera, which included leaves, rhizomes and seeds, and their results showed that the macromolecules obtained from different parts exhibited different biological activities [21]. The rhizome of *P. sibiricum* can also be divided into young rhizome and mature rhizome as extraction method for PsP, and the mature rhizome often is used as the main extraction raw material for PsP [22] in practical production. Li et al. [23] studied the physical and chemical indexes of different parts of *P. sibiricum*, whose results showed that the PsP contents of mature rhizome was the highest, follow by young rhizome. However, the young rhizome of by-product generally is regarded as waste to discard directly after the mature rhizome aged three-to-five years is utilized as the source material to extract PsP in practical production. This has consequently led to a huge wastage of *P. sibiricum* resources. The reason for this is a lack of research on this topic, which could prove that the PsP value of young rhizome is similar to that of mature rhizome, and whether the discarded young rhizome also has similar biological activities to the mature rhizome. It is regrettable that comparative studies about the extraction rate, structure composition and biological activities between young and mature rhizomes from *P. sibiricum* are lacking. Therefore, it is worth exploring whether there are differences in the above aspects between them, and whether the PsP from young rhizome of by-product should not be entirely discarded as waste. This study aimed to compare the abilities of extraction, structure composition and antioxidant ability of PsP between young and mature rhizomes. The comparisons of PsP between young and mature *P. sibiricum* rhizomes were conducted on various aspects, which included extraction rate, molecular weight, monosaccharide composition, infrared spectra, and in vitro antioxidant activity. The purpose of this study is to prove that the PsP value of young rhizome is similar to that of mature rhizome. This can provide a fully theoretical basis for the optimal and comprehensive utilization of *P. sibiricum* resources, and to realize the effect of energy saving and emission reduction of PsP production.

## 2. Materials and Methods

### 2.1. Materials

*P. sibiricum*, that mainly grows in the shades under a tree, was purchased from Beijing Huahongkang Chinese Herbal Medicine Planting Co., Ltd. (Beijing, China). The young and mature rhizomes of our study are two parts of one entire rhizome of *P. sibiricum* that grows in the same place of YanShan mountain.

All chemicals used in this study were analytical grade. The reagents utilized in this study can be found in Table 1.

### 2.2. Main Instruments and Equipment

Allegra x-22 R centrifuge (Beckman coulter, Inc., Brea, CA, USA); SpectraMax I3X Enzyme marker (Molecular Devices Instruments Ltd., San Jose, CA, USA); digital ultrasonic cleaner (Kunshan Ultrasonic Instruments Co., Ltd., Kunshan, China); vortex mixing device ORTEx Genius (IKA, Inc., Staufen, Germany); magnetic stirrers (IKA, Inc., Staufen, Germany); U-3900 spectrophotometer (Hitachi, Ltd., Tokyo, Japan); circulating water multi-purpose vacuum pump (Gongyi Yuhua Instrument Co., Ltd., Zhengzhou, China); freeze dryer (Ningbo Scientz Biotechnology Co., Ltd., Ningbo, China); rotary evaporator (Shanghai xiande experimental instrument Co., Ltd., Shanghai, China); desk centrifuge 5418 (Eppendorf, Inc., Hamburg, Germany); 1100 Series Gel size exclusion chromatograph (Agilent Technologies, CA, USA); 1200 Infinitely high performance liquid chromatography (Agilent Technologies, CA, USA); TENSOR 27 Near-infrared diffuse reflectance spectrometry (Germany BRUKER, Karlsruhe, Germany).

### 2.3. Experimental Technical Process Chart

**Figure 1:**
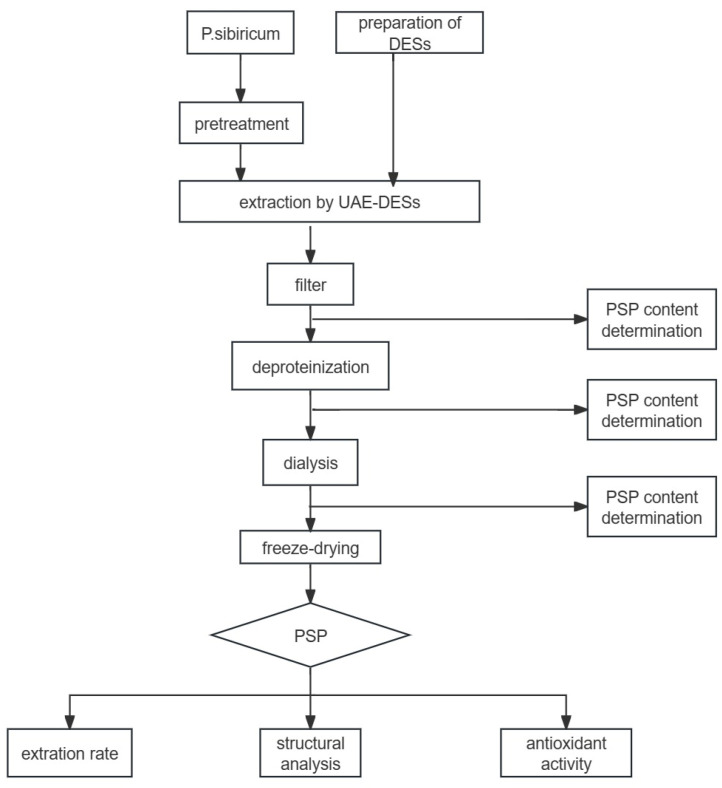
Experimental technical process chart. Abbreviations: DESs, deep eutectic solvents; UAE-DESs, ultrasound-assisted extraction-deep eutectic solvents; PSP, *Polygonatum sibiricum* Polysaccharide.

The specific technical process of this study is shown in Figure 1.

### 2.4. Extraction of PsP 

The distinction between young and mature rhizomes was conducted according to Li et al. [23], and the practical experience of our team in previous research. The PsP extraction was carried out by using the ultrasound-assisted extraction-deep eutectic solvents (UAE-DESs) method, which our team has employed in previous studies [24]. 

#### 2.4.1. Distinguishment of Young and Mature Rhizomes, and DESs Preparation 

The rhizome of *P. sibiricum* was washed and the fibrous rhizome was trimmed. We delineated rhizome into two categories using a 6 mm threshold according to Li et al. [23] and our team’s prior experience: the rhizomes whose diameters were less than 6 mm were classified as young rhizome, while those whose diameters were above 6 mm were classified as mature rhizome. Both young and mature rhizomes of *P. sibiricum* were dried in an oven at 45 °C until a constant weight after they were cut into thin slices. Subsequently, the dried slices were crushed. And then, the *P. sibiricum* powder was stored in a vacuum dryer after it had been screened using an 80 micron mesh sieve. 

Choline chloride and 1,4-butanediol were mixed at a molar ratio of 1:4. The mixture was placed on a magnetic stirrer under conditions of 70 °C and 1000 rpm until a homogeneous solution [25]. Water was then added to produce a 32% aqueous solution, which was fully mixed with the above homogeneous solution on the magnetic stirrer under the same conditions. This process resulted in deep eutectic solvent (DESs) [26,27]. 

#### 2.4.2. Extraction and Purification

After dried *P. sibiricum* powder was added into a centrifuge tube, the DESs was added according into the 4:1 liquid-to-material ratio, and then, the PsP was extracted with ultrasonic assistance for 51 min at 82 w and 80 °C. Then, anhydrous ethanol was added into the centrifuge tube at a liquid-to-mixture ratio of 4:1. After the sample was processed by alcohol precipitation at 4 °C for 12 h, PsP was collected by using centrifugation at 5000 rpm for 15 min [24,28].

The extracts were filtered to remove insoluble residue. After that, the filtrate was further treated by the Sevag method so as to remove proteins. And then, dialysis with a 1000 Da membrane was carried out for two days to remove small molecular substances. The filtrate was further concentrated to 3–4 mL at 45 °C using a rotary evaporator. Finally, the purified PsP was obtained through freeze-drying. 

### 2.5. Chemical Composition Analysis

#### 2.5.1. Determination of PsP

The PsP content was determined by employing 0.2% anthrone-sulfuric acid reagent according to a procedure described by Sun et al. [24]. The 100 μL PsP extract was added into 1.9 mL distilled water, then 8 mL of 0.2% anthrone-sulfuric acid solution was added into them. The mixed solution was conducted in a water bath at 80 °C for 20 min. After cooling, the absorbance value of extract was measured at 582 nm using a UV spectrophotometer. A series of working concentration of Anhydrous D-Glucose (from 15 to 100 µg/mL) were used to plot the analytical curve. The results were expressed as mg of glucose equivalent per gram of extract. The PsP contents before and after protein removal, and that of inside and outside of the dialysis sack after dialysis were determined respectively. The purity and extraction rate of PsP were calculated using the Equation (1).

(1) $$R(\%)=\frac{C\times V\times N}{M}\times 100$$

*R*: extraction rate, C: PsP concentration, V: dilution multiple, N: dilution multiple, M: *P. sibiricum* quality.

#### 2.5.2. Determination of Protein Content in PsP Solution

The content of protein was determined by Coomassie brilliant blue test procedure described by Sun et al. [24]. Before adding 5 mL Coomassie brilliant blue test solution, 100 μL of the extract was added to 0.9 mL of distilled water. After the solution was incubated for 2 min, the absorbance value was measured at 595 nm with a UV spectrophotometer. The standard curve was constructed with bovine serum albumin at the working concentrations of 20 to 100 µg/mL [29,30]. The results were expressed as mg of bovine serum albumin equivalent per gram of extract.

### 2.6. Molecular Weight Determination of PsP 

The PsP was dissolved in 0.2 M ammonium bicarbonate solution, and the supernatant was injected into a Sephadex G-50 (Shanghai yuanye Bio-Technology Co., Ltd., Shanghai, China) column after centrifugation at 10,000 rpm for 10 min. Elution was performed with 0.2 M ammonium bicarbonate, with 10 mL per tube. The phenol-sulfuric acid method was used to measure the concentration of each tube, and gel chromatography was drawn. The peak fractions were collected and concentrated to a small volume by repeated removing ammonium bicarbonate. The PsP liquid sample filtered by membrane was analyzed by high performance gel permeation chromatography (HPGPC) and TSK-gel G3000 PWXL column (Guangzhou Lubex Scientific Instrument, Co., Ltd., Guangzhou, China), and the column temperature was kept at 40 °C. Detection was achieved using a differential refractive index detector, with a flow rate set at 0.5 mL/min and a mobile phase comprising 0.05 M sodium sulfate. The chromatographic column was calibrated utilizing a range of distinct dextran standards to generate a standard curve [31]. 

A linear correlation was established, employing log (Mw) as the x-axis and retention time as the y-axis.

### 2.7. Detection of Monosaccharide Component 

Following the methodology of the literature [32], the samples, ultrapure water (UP) and 4 M trifluoroacetic acid (TFA) were added into the ampoules. Then, the ampoules were vacuum-sealed and placed at 110 °C for 4 h. After the reaction, the mixture was neutralized to pH 7.0, and a hydrolyzed monosaccharide mixture was obtained. Next, the sample with equal volumes and internal standard solution (2-deoxyribose) were thoroughly mixed. 0.3 M NaOH and 0.5 M 1-phenyl-3-methyl-5-pyrazolone (PMP) solution were added to the mixture. The pH of the reaction solution was adjusted to 7.0 after conducting at 70 °C for 1 h. Chloroform was added for repeated extraction, and the final extracted supernatant was filtered and conducted HPLC analysis. In total, 10 kinds of standard monosaccharides, which included D-mannose (Man), D-ribose (Rib), L-sorbose (Sorb), D-glucuronic acid (GlcA), D-galacturonic acid (GalA), D-glucose (Glc), D-galactose (Gal), D-xylose (Xyl), D-arabinose (Ara), and L-fucose (Fuc), were selected.

The HPLC detection parameters were as follows: chromatographic column of Agilent Eclipse XDB-C18 (Agilent Technologies, Inc., Santa Clara, CA, USA) (5 μm, 4.6 × 250 mm), flow rate of 1 mL/min, column temperature of 30 °C, detection wavelength of 254 nm, mobile phase A: 18% acetonitrile solution with triethylamine, mobile phase B: 60% acetonitrile solution with triethylamine. 

### 2.8. Analysis by Infrared Spectroscopy 

A total of 2 mg of PsP powder was mixed with 100 mg of KBr powder, and they were placed into a mortar. The mixture was fully ground, and then was compressed into uniform and transparent particles by a tablet press. Subsequently, the particles were scanned within the range of 4000 to 400 cm^−1^ using Fourier-transform infrared spectrometer. 

### 2.9. In Vitro Antioxidant Activity 

#### 2.9.1. DPPH Radical Scavenging Rate 

The DPPH free radical scavenging ability was evaluated by the method described by Sun et al. [24], which existed small modifications. First, an 80 µmol/L DPPH methanol solution was prepared and stored away from light. Then, the different concentrations (1, 2, 4, 8, 16 mg/mL) of PsP solution were prepared. This experiment included three groups. The PsP sample group contained 30 µL PsP solution and 180 µL DPPH methanol solution, and the control group contained 30µL PsP solution and 180 µL methanol solution, and the blank group was not to zero the instrument, but contained 30 µL distilled water and 180 µL of DPPH methanol solution for comparing with PsP sample group. The mixture of each group was reacted for 30 min away from light in a 96-well plate at room temperature, and the absorbance value was measured at 517 nm using an enzyme marker [33]. A series of vitamin C solutions with different concentration (10, 20, 40, 60, 80 µg/mL) were used as a positive control for comparison. The DPPH (Shanghai Shifeng biological technology Co., Ltd., Shanghai, China) free radical scavenging rate was calculated using the Equation (2).

(2) $$\mathrm{DPPH}\;\mathrm{radical}\;\mathrm{scavenging}\;\mathrm{rate}\;\left(\%\right)=\left(1-\frac{A_{j}-A_{i}}{A_{o}}\right)\times 100$$

A_j_: Absorbance of PsP sample group, A_i_: Absorbance of control group, A_o_: Absorbance of blank group.

According to the above DPPH radical scavenging rate, the IC50 value of PsP, which refers to the concentration of PsP solution when the radical scavenging rate is 50%, could be calculated by the dose-response relationship between PsP concentration and DPPH radical scavenging rate. 

#### 2.9.2. ABTS Radical Scavenging Rate

The ABTS radical scavenging activity of PsP was determined according to the method of Sun et al. [24]. A 7 mmol/L ABTS methanol solution was mixed with a 2.45 mmol/L potassium persulfate solution as a 1:1 ratio to generate the ABTS free radicals. The mixture was allowed to react at 4 °C for 24 h. Then, the solution was further diluted with methanol until the absorbance reached a value of 0.7 ± 0.05 at a wavelength of 734 nm. The various concentrations (1, 2, 4, 8, 16 mg/mL) of the PsP solution were prepared. This experiment included three groups. The PsP sample group contained 20 µL PsP solution and 180 µL ABTS methanol solution, and the control group contained 20µL PsP solution and 180 µL methanol solution, and the blank group was not to zero the instrument, but contained 20 µL distilled water and 180 µL of ABTS methanol solution for comparing with PsP sample group. The mixtures were incubated in the dark at room temperature for 10 min, and the absorbance was measured at 734 nm by an enzyme marker. A series of vitamin C solutions of 10 to 80 µg/mL were used as positive control [34]. Finally, the ABTS (Aladdin, Inc., Shanghai, China) free radical scavenging rate was calculated using the Equation (3).

(3) $$\mathrm{ABTS}\;\mathrm{radical}\;\mathrm{scavenging}\;\mathrm{rate}\;\left(\%\right)=\left(1-\frac{A_{j}-A_{i}}{A_{o}}\right)\times 100$$

A_j_: Absorbance of PsP sample group, A_i_: Absorbance of control group, A_o_: Absorbance of blank group.

According to the above ABTS radical scavenging rate, the IC50 value of PsP, which refers to the concentration of PsP solution when the radical scavenging rate is 50%, could be calculated by the dose-response relationship between PsP concentration and ABTS radical scavenging rate.

#### 2.9.3. Total Oxyradical Scavenging Ability

A 4.0 mL of a phosphorus molybdenum test solution (composed of 0.6 mol/L H_2_SO_4_, 28 mmol/L Na_3_PO_4_, and 4 mmol/L H_8_MoN_2_O_4_, which were mixed in a volume ratio of 1:1:1) was added to 10 mL volumetric flask. Then, the PsP solutions with various concentrations (1, 2, 4, 8, 16 mg/mL) were prepared and 0.4 mL PsP solution of each concentrations was transferred into volumetric flasks. The solutions were thoroughly shaken and heated in a water bath at 95 °C for 1.5 h. After cooling to room temperature, the absorbance value of each solution was measured using UV spectrophotometer analysis at 695 nm. Distilled water was used as the reference solution, and a series of vitamin C solutions (31.25, 62.5, 125, 250, 500 µg/mL) were used as the positive control [35]. The total antioxidant scavenging ability was determined based on the absorbance values, with higher values indicating a stronger antioxidant activity of the sample.

#### 2.9.4. In Vitro Catalase Activity Determination 

We allocated the catalase solution with an activity unit of 200 U/mL according to a certain proportion, and prepared different concentrations (1, 2, 4, 8, 16 mg/mL) of PsP solution simultaneously. The working solution was incubated at 37℃ for 10 min. Next, 1 mL working solution was transferred into a 1 mL quartz colorimetric plate. Then, a 35 µL PsP solution was added and mixed for 5 s. The initial absorbance value (A1) and the absorbance value (A2) after 1 min were immediately measured at 240 nm at room temperature [36]. The difference in absorbance values (∆A = A1 − A2) was calculated, and the catalase activity (U/mL) was calculated using the Equation (4).

(4) CAT(U/mL)=678×ΔA

*CAT*: catalase; ∆A: the difference in absorbance values.

### 2.10. Statistical Analysis

The independent sample t-test was utilized to analyze the single-factor experiment. A bilateral *p*-value less than 0.05 was considered statistically significant. Each experiment was replicated a minimum of four times, and the data were presented as mean ± standard deviation (M ± SD). Statistical analysis was performed using SPSS 27.0 (IBM, Armonk, NY, USA), and statistical graphs were produced with Origin 2022 (Origin Lab Inc., Hampton, MA, USA) and Microsoft Excel 2010.

## 3. Results

### 3.1. Comparisons of PsP Extraction Rates and the PsP Contents at Each Extraction Stage between Young and Mature Rhizomes 

The PsP extraction rates of young and mature rhizomes were (33.88 ± 1.95)% and (45.08 ± 1.92)%, respectively, as using UAE-DESs method. The PsP extraction rate from young rhizome were less than that from mature, which was significant at the *p* = 0.05 level. 

The PsP contents of young and mature rhizomes at each extraction stage were shown in Table 2. The protein contents of young and mature rhizomes were (1.42 ± 0.31)% and (1.08 ± 0.25)%, respectively, without significant difference between the young and mature rhizomes. The PsP purifies of young and mature rhizomes were (98.24 ± 0.47)% and (98.28 ± 0.30)%, respectively, without significant difference between the young and mature rhizomes, too. These results indicated that there were comparability between young and mature rhizomes in further comparison of in vitro antioxidant ability of PsP. The results are shown in Table 2.

### 3.2. Comparison of Molecular Weight of PsP between Young and Mature Rhizomes

**Figure 2:**
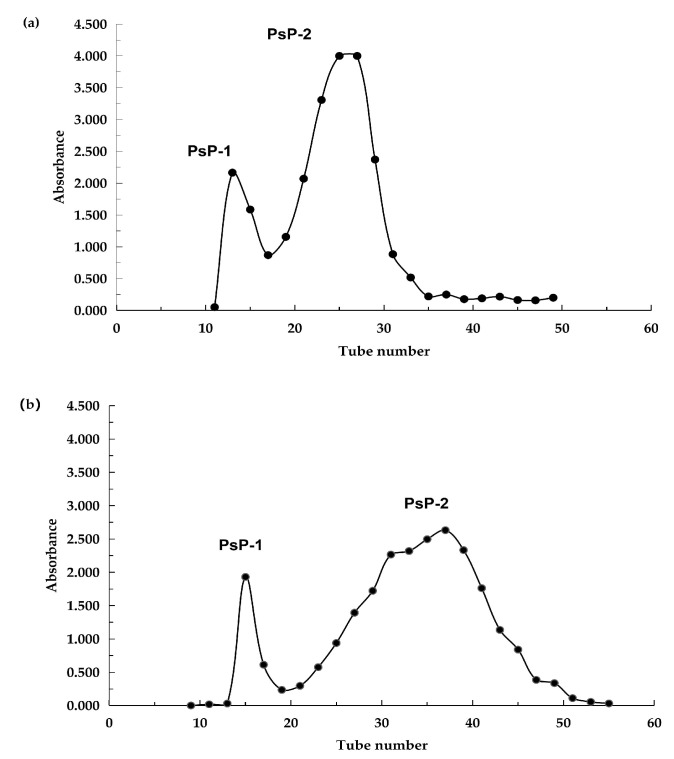
Elution curve of PsP. (**a**) Elution curve of PsP from young rhizome on Sephadex G-50 column. (**b**) Elution curve of PsP from mature rhizome on Sephadex G-50 column [24]. Abbreviations: PsP, *Polygonatum sibiricum* Polysaccharide.

We obtained the separated components of PsP by Sephadex G-50 column. The results appear in the Figure 2 by the elution curve. For both young and mature rhizomes, PsP-2 was the main component of PsP, which is based on the proportionality between peak area and PsP content.

**Figure 3:**
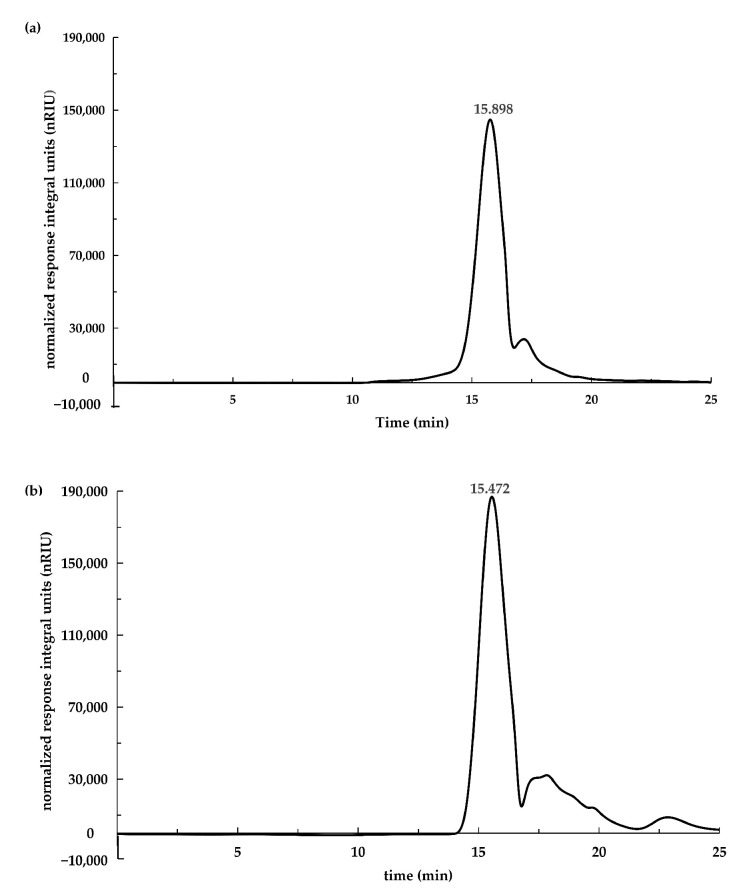
Molecular weight distribution of PsP. (**a**) Molecular weight distribution of PsP from young rhizome. (**b**) Molecular weight distribution of PsP from mature rhizome [24]. Abbreviations: PsP, *Polygonatum sibiricum* Polysaccharide.

Figure 3 depicts the HPGPC results of PsP-2 extracted from young and mature rhizomes. Table 3 presents the retention times and molecular weights of PsP-2 from young and mature rhizomes. The retention times of PsP-2 observed from young and mature rhizomes were 15.898 min and 15.472 min, corresponding to molecular weights of 2100 Da and 4600 Da, respectively.

### 3.3. Composition of Monosaccharide Compositions of PsP between Young and Mature Rhizomes

**Figure 4:**
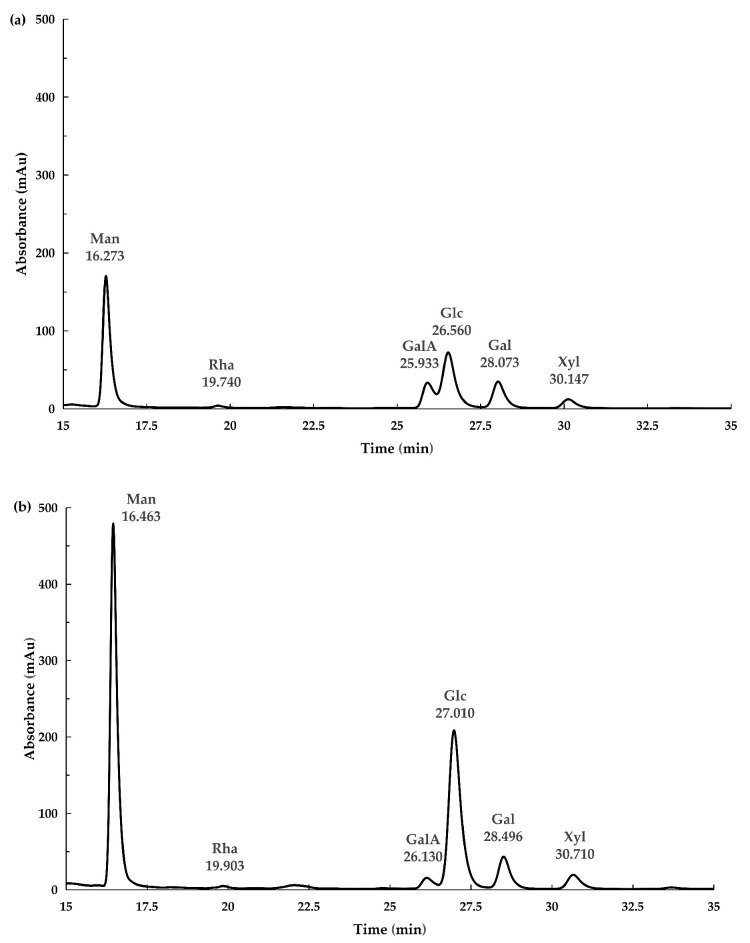
Monosaccharide composition of PsP. (**a**) Monosaccharide composition of PsP from young rhizome. (**b**) Monosaccharide composition of PsP from mature rhizome. Note: The monosaccharides are Man, Rha, GalA, Glc, Gal and Xyl in chronological order, successively. Abbreviations: PsP, *Polygonatum sibiricum* Polysaccharide; Man, mannose; Rha, rhamnose; GalA, galactose acid; Glc, glucose; Gal, galactose; Xyl, xylose.

The HPLC chromatograms presented monosaccharide compositions of PsP from young and mature rhizomes in Figure 4. The findings showed that the PsP of both young and mature rhizomes were composed of six kinds of monosaccharides: mannose (Man), rhamnose (Rha), galactose acid (GalA), glucose (Glc), galactose (Gal) and xylose (Xyl). The molar ratios of monosaccharide composition for PsP from young and mature rhizome were introduced in Table 4. 

### 3.4. Comparison of Infrared Spectroscopic Analysis of PsP between Young and Mature Rhizomes

**Figure 5:**
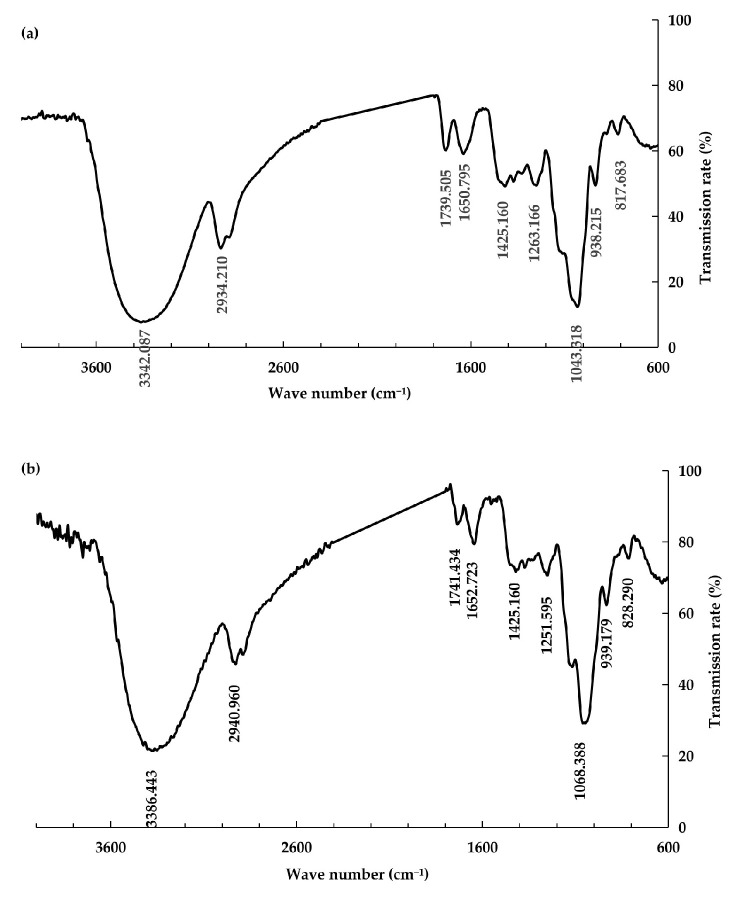
Infrared spectrum of PsP. (**a**) Infrared spectrum of PsP from young rhizome. (**b**) Infrared spectrum of PsP from mature rhizome. Abbreviations: PsP, *Polygonatum sibiricum* Polysaccharide.

The results of infrared spectrum of PsP extracted from both young and mature rhizomes are shown in Figure 5. There were similar structure characteristics in PsP between young and mature rhizomes. The broad and smooth absorption peaks, which were observed at 3342.087 cm^−1^ and 3386.443 cm^−1^ for the PsP of both young and mature rhizomes, were attributed to the stretching vibrations of intramolecular or intermolecular -OH groups within the polysaccharide molecules. Peaks at 2934.210 cm^−1^ and 2940.960 cm^−1^ corresponded to the stretching vibrations of -CH_2_ groups. Peaks around 1263.166 cm^−1^ and 1251.595 cm^−1^ were produced by the symmetric and asymmetric stretching vibrations of C=O and free carboxylic acids, which showed the presence of uronic acid residues, and which indicated PsP of both young and mature rhizomes were acidic polysaccharide. Peaks at 1650.795 cm^−1^ and 1652.723 cm^−1^ were attributed to C=O stretching vibrations, and those around 1400 cm^−1^ corresponded to C-H bending vibrations. Peaks observed at 1043.318 cm^−1^ and 1068.388 cm^−1^ represented typical absorption peaks of furanose sugars, which indicated the presence of furanose sugars in the samples. The absorption peaks around 900 cm^−1^ indicated the presence of β-glycosidic bonds in the samples [37]. The results showed the PsP from young and mature rhizomes had characteristic structure of polysaccharide and belonged to acidic polysaccharide.

### 3.5. Comparisons of Antioxidant Ability of PsP between Young and Mature Rhizomes

#### 3.5.1. Comparison of DPPH and ABTS Scavenging Ability Analysis 

**Figure 6:**
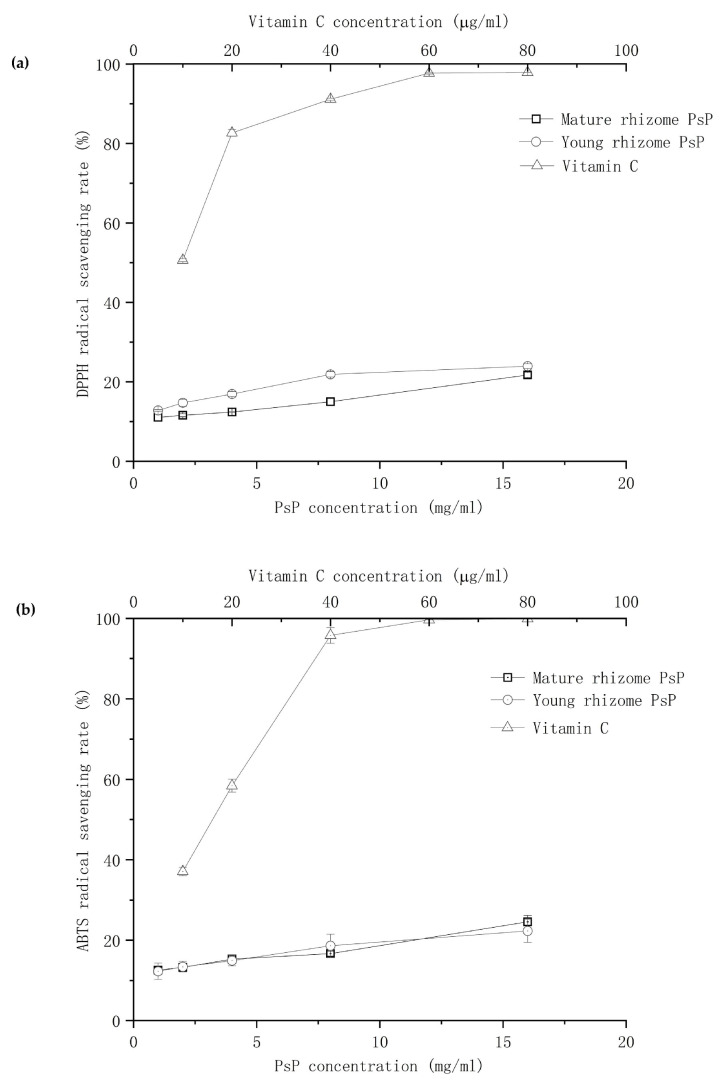
Results of DPPH and ABTS scavenging rate. (**a**) Results of DPPH scavenging rate. (**b**) Results of ABTS scavenging rate. Abbreviations: PsP, *Polygonatum sibiricum* Polysaccharide.

The results of the DPPH and ABTS scavenging ability experiments showed that the scavenging effect of PsP from young and mature rhizomes on radicals increased gradually with concentrations ranging from 1 to 16 mg/mL, with a more obvious effect observed at higher concentrations. The difference of DPPH radical scavenging ability of PsP between young and mature rhizomes was significant (*p* < 0.05), and the DPPH radical scavenging ability of PsP from young rhizome was significantly higher than that from mature rhizome. However, there was no significant difference in the ABTS scavenging ability of PsP between young and mature rhizomes. The results of IC50 values of DPPH and ABTS scavenging ability are shown in Table 5, and the DPPH and ABTS scavenging abilities are shown in Figure 6.

#### 3.5.2. Comparison of Total Oxyradical Scavenging Ability Analysis 

**Figure 7:**
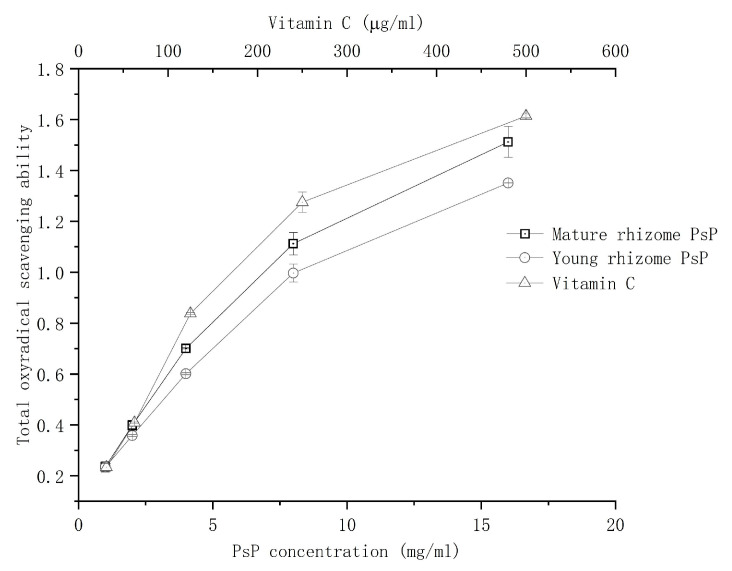
Results of total oxyradical scavenging ability. Abbreviations: PsP, *Polygonatum sibiricum* Polysaccharide.

Total antioxidant ability experiments demonstrated a gradual enhancement in antioxidant activity with increasing PsP concentrations (1–16 mg/mL) of both young and mature rhizomes. The difference in total oxyradical scavenging rate of PsP between young and mature rhizomes was significant (*p* < 0.05), and the total oxyradical scavenging ability of young rhizome was lower than that of mature rhizome. The results are shown in Figure 7.

#### 3.5.3. Comparison of In Vitro Catalase Activity

**Figure 8:**
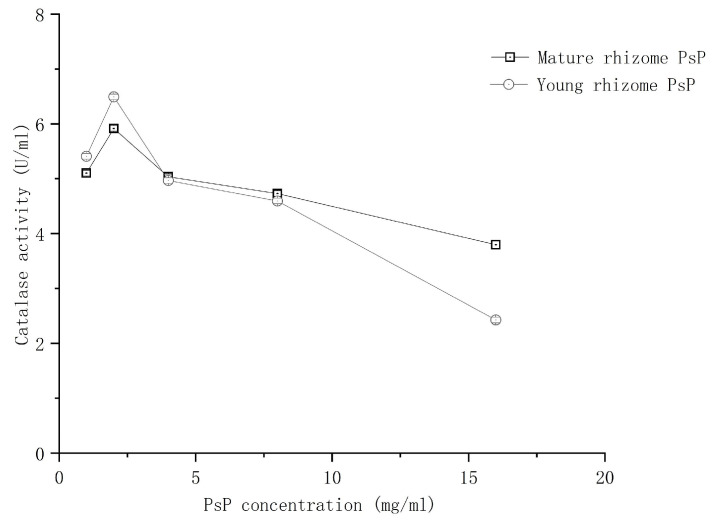
Results of in vitro catalase activity. Abbreviations: PsP, *Polygonatum sibiricum* Polysaccharide.

Figure 8 shows that the in vitro antioxidant activity of catalase was enhanced by PsP from young rhizome within a concentration range from 1 to 2 mg/mL. Then, the promoting effect of PsP on the catalase activity decreased after 2 mg/mL. The antioxidant activity of catalase in the young rhizome PsP was consistent with that of the PsP of mature rhizome. There was no statistically significant difference between the young rhizome and the mature rhizome PsP. In addition, the PsP of young rhizome was higher than that of mature rhizome in catalase activity within a concentration range from 1 to 4 mg/mL, and it was lower than that of mature rhizome after 4 mg/mL, without significant difference between the above two stages. 

## 4. Discussion

This study compared the extraction rate, structure composition and antioxidant capacity of PsP between young and mature rhizomes of *P. sibiricum*. The extraction rate of PsP from young rhizome was similar to that from mature rhizome. The structure composition of PsP extracted from young rhizome was no different with that of PsP from mature rhizome basically. In addition, the PsP of young rhizome appeared to exhibit similar biological activity to that of the mature rhizome, which indicated more advantages in DPPH free radical scavenging ability than the PsP of mature rhizome. This study proved the young rhizome of by-product is also worthy, and should not be discarded directly, which would avoid the unnecessary waste of *P. sibiricum* resources. The results provided a reference basis for comprehensive utilization of *P. sibiricum* resources in PsP food industry production.

The UAE-DESs method performs better than conventional extraction methods, which had been proved by previous studies of our team and other teams [24,38,39]. Given that the young and mature rhizomes of *P. sibiricum* were extracted by this same method, this could ensure the comparability of the PsP between young and mature rhizomes.

Li et al. [23] also analyzed the extraction rate among different parts of *P. sibiricum*, with their results showing that the difference in PsP contents between young and mature rhizomes was not significant, while our results indicated that there was a significant difference in PsP contents between the two kinds of rhizomes. The reason might be that different growth environments of *P. sibiricum* can affect the PsP contents of different parts. In addition, the classification threshold of the young and mature rhizomes is different between our method and their method. Currently, there is no objective standard of classification threshold for young and mature rhizomes. In our study, we distinguished young and mature rhizomes using 6 mm diameter according to previous studies and our team’s experience. It was possible that Li et al. [23] adopted more small classification threshold to distinguish young and mature rhizomes, which led to the difference between ours and theirs. 

One study revealed a difference in antioxidant capacity among different parts of Viburnum opulus (fruit, flower and bark) [40]. Liu et al. [41] reported one comparison of immune effects among different parts of Radix Astragali, and other some studies also reported the comparison of pharmacological effects among different parts of Eucommia ulmoides [42]. The results all showed that polysaccharides in different parts from plants had different effects, and subsequently whether the PsP effects from young and mature rhizomes of *P. sibiricum* also exhibit differences. At present, the PsP from mature rhizome has been demonstrated by numerous studies. For instance, one study revealed that PsP of mature rhizome, which played an antioxidant role, could inhibit the production of oxygen free radicals and strengthen the scavenging ability of oxygen free radicals in the brain [43]. Another study showed that PsP of mature rhizomes could effectively scavenge hydroxyl radicals, and inhibit the production of lipid peroxide in liver and the rupture of erythrocyte membrane caused by hydroxyl radicals in rabbits [44]. The above studies have provided an important scientific basis for developing PsP from mature rhizome in the field of health products and medicine. Although Li et al. [23] studied the physical and chemical indexes of different parts of *P. sibiricum*, their study did not report the difference in biological activity between young and mature rhizomes of *P. sibiricum*. Therefore, our study improved the study field of PsP biological activity of different rhizomes of *P. sibiricum*.

Our results showed that the molecular weight of the young rhizome PsP (2100 Da) was lower than that of the mature rhizome PsP (4600 Da). On the one hand, the reason might be that the content of monosaccharide with large molecular weight (such as Man and Gal) in the PsP from mature rhizome is higher than that in the PsP from young rhizome, which might lead to the PsP molecular weight of mature rhizome being larger. On the other hand, the growth time of young rhizome is shorter, resulting in more monosaccharide and oligosaccharide, and less PsP content. This explanation had been proved by our experiment result of “Section 3.1”. In this experiment, the crude PsP solution was separated into monosaccharide, oligosaccharide and PsP during the purification process with dialysis, whose principle is the saccharides of small molecules are excluded through semi-permeable membranes [45], and the monosaccharide and oligosaccharide diffuse into water outside of the semi-permeable membranes. Therefore, the PsP molecular weight of the young rhizome was less than that of the mature rhizome. In addition, the young rhizome of *P. sibiricum* is in a growth stage, requiring significant energy and nutrients to support its rapid growth. Therefore, the young rhizome tends to utilize monosaccharide and oligosaccharide directly as energy sources rather than convert them into PsP. The content of monosaccharide that is used to synthesize PsP is low, so the PsP chain is shorter in young rhizome. Therefore, the molecular weight of PsP from young rhizome was lower than that from mature rhizome.

To our surprise, the results showed that the molar ratios of other monosaccharides except glucose in PsP from young rhizomes were higher than those from mature rhizomes. These results indicated that the molar ratios of monosaccharides continuously decreased during the growth process of *P. sibiricum* rhizome. In young rhizome, the contents of six kinds of monosaccharides were similar, while both Glc and Man contents were obviously higher than the other four kinds of monosaccharides in mature rhizome. The increase in Glc content directly led to the decrease in molar ratios of the other five kinds of monosaccharides in the mature rhizome. In addition, the molar ratios of GalA and Xyl in young rhizome were obviously higher than those in mature rhizome, as shown by the above results. A study [46] indicated that monosaccharides with different molar ratios could affect the biological activity of polysaccharides, and polysaccharides with higher molar ratios of GalA and Xyl could enhance DPPH free radicals scavenging ability. The in vitro antioxidant experiment of our study showed the similar result that the DPPH free radical scavenging ability of the PsP from young rhizome was higher than that of mature rhizome, which reason might be the molar ratios of GalA and Xyl in PsP from young rhizome were higher than those from mature rhizome. 

Our study showed that there were some similar properties in functional groups of PsP between young and mature rhizomes of *P. sibiricum*. The PsP infrared spectra of young and mature rhizomes exhibited similar peak positions. Referring to the research results of Chen et al. [47], it was considered that the PsP of both young and mature rhizomes contained O-H groups, C-H groups and C=O groups. The above characteristic absorption peaks proved the PsP of young rhizome has a similar polysaccharide nature to the mature rhizome. In addition, the absorption peaks of furanose sugars and uronic acid constituents of PsP of young rhizome was similar to those of mature rhizome. 

The result of PsP purity showed the PsP purity values of both young and mature rhizomes exceeded 98%, and there was no significant difference in PsP purity values between them. Therefore, the result proved there was comparability between young and mature rhizomes in further comparison of in vitro antioxidant ability of PsP.

Although in vitro antioxidant experiments present limitations, which cannot fully reflect the in vivo true conditions of the body, the blind in vivo antioxidant experiments not only waste enormous human resources, but also require enormous financial resources. Therefore, in vitro antioxidant experiments are still regarded as a basic and independent part of evaluating antioxidant activity [24]. Many scholars have employed in vitro antioxidant experiments, which can be applied independently, to serve as a necessary exploration of subsequent in vivo antioxidant experiments [33]. Therefore, our study adopted four different indexes of in vitro antioxidant experiments to analyze and compare the antioxidant activity of PsP between young and mature rhizomes. This intended to more comprehensively reflect the antioxidant activity of PsP of young rhizome to the greatest extent, and provide research evidence on the feasibility of that in subsequent in vivo antioxidant experiments. 

Previous studies have proved that there was a certain linear relationship between DPPH free radicals scavenging ability and the PsP concentration [18], which were consistent with ours. In addition, our study proved that the DPPH free radicals scavenging ability of the PsP of young rhizome was significantly higher than that of mature rhizome. One reason is mentioned in the above discussion. The other reason might be, the polysaccharide, that can donate more hydrogen atoms or electrons, can neutralize more free radicals by providing more hydrogen atoms or electrons, and thus better inhibit the oxidation process and reduce oxidative stress [48]. Therefore, the reason that the DPPH free radicals scavenging ability of the PsP of young rhizome was higher than that of mature rhizome, might be that the young rhizome possesses more hydrogen atoms or electrons. According to our results and the above analysis, the young rhizome should be preferred but not be discarded directly if we pay more attention to high DPPH free radicals scavenging ability. 

The result of total antioxidant ability showed that the PsP total antioxidant activity of young rhizome was lower than that of mature rhizome. The results can be explained by the fact that the quantity and density distribution of special functional groups might be different between young and mature rhizomes, although the PsP of young rhizome possesses the same kinds of functional groups as that of the mature rhizome as seen in the results of “Section 3.4”. The hydroxyl and carboxyl groups of polysaccharide, which are crucial for the total antioxidant ability, can directly interact with phosphorus molybdenum reagent and promote their reduction. So, the quantity and density distribution of the above two functional groups can directly influence the total antioxidant ability of the polysaccharide [31]. Therefore, it is possible that the PsP of young rhizome possesses less quantity and lower density distribution of hydroxyl and carboxyl groups according to our result. In addition, the PsP total antioxidant ability of both young and mature rhizomes gradually increased with an increasing PsP concentration ranging from 1 to 16 mg/mL, which is consistent with studies of others [5]. Therefore, the PsP total antioxidant ability of young rhizome is similar to that of mature rhizome, which proved young rhizome is still worthy to play total antioxidant ability. The above results can avoid unnecessary waste and realize comprehensive utilization of the *P. sibiricum* resource more effectively.

There were no significant differences between young and mature rhizomes in both ABTS free radicals scavenging ability and antioxidant activity of catalase. The ABTS free radicals scavenging abilities of the PsP in both young and mature rhizomes revealed a certain linear relationship with concentrations, which further indicated that the PsP of young rhizome has the same ABTS free radicals scavenging ability as that of the mature rhizome. Therefore, it is worthwhile to extract PsP from young rhizomes, so as to realize the optimal utilization of *P. sibiricum* resources. The PsP of both young and mature rhizomes could promote the catalase activity within a certain concentration range. The results indicated that the lower PsP concentrations, and the PsP of young rhizomes can provide a more suitable environment to improve the catalase activity by stabilizing its structure. This phenomenon is attributed to the fact that different pH environments can generate different effects on catalase activity [37], and different PsP concentrations and the PsP of young and mature rhizomes can generate different pH environments as the characteristic of acidic PsP. Therefore, the low concentrations of PsP in young rhizomes should be preferred if we are to pay more attention to high catalase activity, which can promote the application of the PsP as a natural dietary supplement in the food industry. 

The polysaccharide is a heterogeneous biomolecule, which not only can exist alone, but also combine with other biological macromolecules to form new substances, such as glycoproteins [5]. In the process of extracting PsP, the crude PsP of both young and mature rhizomes, which includes polysaccharide and polysaccharide combined with protein [26], was deproteinized under the same number of times until there was no protein in the PsP solution. According to the result of “Section 3.1”, the difference in value of PsP content before and after deproteinization of the young rhizome was lower than that of the mature rhizome. So, it is possible that the glycoprotein content of the young rhizome might be lower than that of the mature rhizome. This opinion needs further confirmation through subsequent research, which might provide a new study direction for researchers. 

We conducted a comprehensive comparative study of the PsP extracted from the young and mature rhizomes of *P. sibiricum*. Although the PsP content of young rhizome is lower than that of mature rhizome, it still has similar structural composition and in vitro antioxidant ability to that of mature rhizome. In addition, the PsP of young rhizome even has more advantages in DPPH free radical scavenging ability than that of mature rhizome. These findings suggest that the utilization of the young rhizome of by-product in *P. sibiricum* is worthy if rationally use the young rhizome, which can provide a theoretical basis for the comprehensive application of young rhizome in the food industry. 

## 5. Conclusions

This study obtained the conclusions that the PsP of young rhizomes exhibited similar extraction rate, structural composition and in vitro antioxidant ability to that of mature rhizomes. Moreover, the molar ratios of GalA and Xyl in PsP of young rhizomes were found to be higher than those in mature rhizomes. Additionally, the PsP of young rhizomes indicated enhanced DPPH free radicals scavenging ability than that of mature rhizomes through an analysis of the PsP in young rhizome by-product. Therefore, the discarded young rhizome of by-product from *P. sibiricum* still has a certain utilized value, which should not be regarded as waste without consideration. Our findings can provide a reference basis for reducing the waste and comprehensive utilization of *P. sibiricum* resources, which can further avoid the unnecessary waste in PsP food industry production. This study improved the research field of PsP and promoted the PsP application of young rhizome as a natural dietary supplement in the food industry. In the follow-up study, we will study the PsP effect and mechanism of young rhizome in delaying sarcopenia obesity (SO) based on the previous research of our team [24], and explore further the comprehensive value of the young rhizome from *P. sibiricum* in the food industry.

## Figures and Tables

**Table 1:** Reagents used in this research.

The Name of Product	The Code of Product	Company (City, State, Country)
2,2-diphenyl-1-picrylhydrazyl (DPPH)	BSF220911	Shanghai Shifeng biological technology Co., Ltd. (Shanghai, China)
2,20-azino-bis(3-ethylbenzothiazoline-6- sulfonic acid) (ABTS)	221899	Aladdin, Inc. (Shanghai, China)
Bovine serum albumin (BSA)	WXBC7961V	Sigma Co., Ltd. (St. Louis, MO, USA)
D-Glucose anhydrous-RM	RMT13890	Beijing Manhagebio-tech, Co., Ltd. (Beijing, China)
Coomassie brilliant blue G-250	727Y032	Beijing Solarbio life science, Inc. (Beijing, China)
Catalase (CAT) Assay Kit	20221008	Beijing Solarbio life science, Inc. (Beijing, China)
Catalase	1129Y021	Beijing Solarbio life science, Inc. (Beijing, China)
TSK-gel G3000 PWXL column	0008033	Guangzhou Lubex Scientific Instrument, Co., Ltd. (Guangzhou, China)
Eclipse XDB-C18	7995118-585	Agilent Technologies, Inc. (Santa Clara, CA, USA)
Sephadex G-50	#9048-71-9	Shanghai yuanye Bio-Technology Co., Ltd. (Shanghai, China)

**Table 2:** The PsP contents from young and mature rhizomes at each extraction stage.

Material (mg)	Extraction Yield mg (%)	Deproteinization mg (%)	Inside of Dialysis Bag mg (%)	Outside of Dialysis Bag mg (%)	End Product mg (%)	Purity %
Young rhizome PsP (1250)	423.50 ± 24.32 (33.88 ± 1.95)%	136.13 ± 10.94 (10.89 ± 0.64)%	33.67 ± 2.69 (2.69 ± 0.22)%	87.79 ± 2.63 (7.02 ± 0.21)%	37.42 ± 2.48 (2.99 ± 0.20)%	(98.24 ± 0.47)% *
Mature rhizome PsP (1250)	563.46 ± 23.96 (45.08 ± 1.92)%	242.61 ± 10.69 (19.38 ± 0.86)%	83.95 ± 4.78 (6.71 ± 0.38)%	137.29 ± 1.77 (10.98 ± 0.22)%	67.83 ± 2.14 (5.43 ± 0.17)%	(98.28 ± 0.30)% *

Note: *, No significant difference with *p* > 0.05.

**Table 3:** Molecular weight data analysis of PsP.

Sample	Retention Times (min)	Mw (Da)
Young rhizome PsP	15.898	2100
Mature rhizome PsP	15.472	4600

Abbreviations: PsP, *Polygonatum sibiricum* Polysaccharide.

**Table 4:** Monosaccharide composition data analysis of PsP (molar ratio).

Sample	Man	GlcN	Rha	GlcNAc	GlcA	GalA	Glc	Gal	Xyl	Fuc
Young rhizome PsP	1.34	0.00	0.04	0.00	0.00	0.26	1.00	0.39	0.15	0.00
Mature rhizome PsP	1.27	0.00	0.01	0.00	0.00	0.04	1.00	0.17	0.09	0.00

Abbreviations: PsP, *Polygonatum sibiricum* Polysaccharide; Man, mannose; GlcN, glucosamine; Rha, rhamnose; GlcNAc, N-acetylglucosamine; GlcA, glucuronic acid; GalA, galactose acid; Glc, glucose; Gal, galactose; Xyl, xylose; Fuc, fucose.

**Table 5:** IC50 value for extracts and vitamin C.

Type of Experiment	Young Rhizome PsP (mg/mL)	Mature Rhizome PsP (mg/mL)	Vitamin C (μg/mL)
Scavenging rate of DPPH radical	38.89	54.77	9.86
Scavenging rate of ABTS radical	56.58	49.15	16.49

Abbreviations: PsP, *Polygonatum sibiricum* Polysaccharide.

## Data Availability

The data presented in this study are available on request from the corresponding author. The data are not publicly available due to privacy restrictions.

## Abbreviations

*P. sibiricum*	*Polygonatum sibiricum*
PsP	*Polygonatum sibiricum* Polysaccharide
DESs	Deep eutectic solvents
UAE-DESs	Ultrasound-assisted extraction-deep eutectic solvents
DPPH	2,2-diphenyl-1-picrylhydrazyl
ABTS	2,20-azino-bis(3-ethylbenzothiazoline-6-sulfoniacid)
BSA	Bovine serum albumin
UP	Ultrapure
TFA	Trifluoroacetic acid
PMP	1-phenyl-3-methyl-5-pyrazolone
SO	Sarcopenia obesity
